# Research Defence Society

**Published:** 1910-01-01

**Authors:** Sydney Holland, F. M. Sandwith, Stephen Paget

**Affiliations:** President; Chairman of Committee; Hon. Treasurer; Hon. Secretary


### Editor's Letter-Box.

### RESEARCH DEFENCE SOCIETY.

*To the Editor of* The Hospital.

Sir,—In view of the General Election, we desire to call the attention of all Parliamentary candidates to the work of the Research Defence Society. It was founded in January, 1908, to make generally known the facts as to experiments on animals in this country, and the regulations under which they are conducted; the immense importance of such experiments to the welfare of mankind; and the great saving of human life and health which is already due to them. We hope that all candidates for Parliament, who may desire to acquaint themselves with these facts, will communicate with the Hon. Secretary, Research Defence Society, 70 Harley Street, W. He will be happy to supply them with literature, and to answer all inquiries.

We would also remind them that the Royal Commission, though it has published all the evidence, has not yet published its final Report and recommendations to Government; and we hope that those candidates who may be unable, at present, to declare themselves in sympathy with our Society, will at least preserve an open mind, till the Report has been published.

We remain, sir,
Yours faithfully,
Cromer, President.
Sydney Holland, Chairman of Committee.
F. M. Sandwith, Hon. Treasurer.
Stephen Paget, Hon. Secretary.

